# Seasonal distribution of anti-malarial drug resistance alleles on the island of Sumba, Indonesia

**DOI:** 10.1186/1475-2875-8-222

**Published:** 2009-09-29

**Authors:** Puji BS Asih, William O Rogers, Agustina I Susanti, Agus Rahmat, Ismail E Rozi, Mariska A Kusumaningtyas, Krisin, Sekartuti, Rita M Dewi, Farah N Coutrier, Awalludin Sutamihardja, Andre JAM van der Ven, Robert W Sauerwein, Din Syafruddin

**Affiliations:** 1 Eijkman Institute for Molecular Biology, Diponegoro 69, Jakarta 10430, Indonesia; 2 Parasitic Diseases Program, Naval Medical Research Unit #2, Komp. P2P/PLP-LITBANGKES, Jl. Percetakan Negara No. 29, Jakarta Pusat 10560, Indonesia; 3 Department of Biomedicine and Pharmacology, National Institute for Health Research and Development, Jalan Percetakan Negara 29, Jakarta Pusat, 10560, Indonesia; 4 Department of Internal Medicine, Radboud University Nijmegen Medical Centre, Nijmegen, The Netherlands; 5 Department of Medical Microbiology, Radboud University Nijmegen Medical Centre, Nijmegen, The Netherlands; 6 Department of Parasitology, Faculty of Medicine, Hasanuddin University, Makasar, Indonesia

## Abstract

**Background:**

Drug resistant malaria poses an increasing public health problem in Indonesia, especially eastern Indonesia, where malaria is highly endemic. Widespread chloroquine (CQ) resistance and increasing sulphadoxine-pyrimethamine (SP) resistance prompted Indonesia to adopt artemisinin-based combination therapy (ACT) as first-line therapy in 2004. To help develop a suitable malaria control programme in the district of West Sumba, the seasonal distribution of alleles known to be associated with resistance to CQ and SP among *Plasmodium falciparum *isolates from the region was investigated.

**Methods:**

*Plasmodium falciparum *isolates were collected during malariometric surveys in the wet and dry seasons in 2007 using two-stage cluster sampling. Analysis of *pfcrt*, *pfmdr1*, *pfmdr1 *gene copy number, *dhfr*, and *dhps *genes were done using protocols described previously.

**Results and Discussion:**

The 76T allele of the *pfcrt *gene is nearing fixation in this population. *Pfmdr1 *mutant alleles occurred in 72.8% and 53.3%, predominantly as 1042D and 86Y alleles that are mutually exclusive. The prevalence of amplified *pfmdr1 *was found 41.9% and 42.8% of isolates in the wet and dry seasons, respectively. The frequency of *dhfr *mutant alleles was much lower, either as a single 108N mutation or paired with 59R. The 437G allele was the only mutant *dhps *allele detected and it was only found during dry season.

**Conclusion:**

The findings demonstrate a slighly higher distribution of drug-resistant alleles during the wet season and support the policy of replacing CQ with ACT in this area, but suggest that SP might still be effective either alone or in combination with other anti-malarials.

## Background

The most widely used anti-malarial drugs, chloroquine (CQ) and sulfadoxine-pyrimethamine (SP), are failing at an accelerating rate in most endemic countries, including Indonesia. In response to this situation the World Health Organization has recommended artemisinin-based combination therapy (ACT), wherein an artemisinin derivative is paired with a second partner anti-malarial drug. This policy, however, has several limitations in resource-poor settings where diagnostic facilities are challenged by availability, cost, compliance and requirements for proper monitoring [1]. In addition, the rapid spread of resistance to currently available anti-malarials is limiting options for ACT partner drugs.

The molecular mechanisms underlying resistance to anti-malarial drugs have been investigated. Several single nucleotide polymorphisms (SNPs) in a number of *Plasmodium falciparum *genes have been associated with resistance to CQ [2] and SP [3]. In addition, amplification of the *Plasmodium falciparum multi-drug resistance 1 *(*pfmdr1*) gene has been associated with mefloquine resistance [4].

In Indonesia CQ, SP and primaquine have been the most widely used anti-malarial drugs. The drugs are readily available but diagnostic facilities are poor, and as a result their use is often inappropriate, leading to the increased spread of resistance. Resistance to CQ was first reported in 1975, resistance to SP appeared in 1978, and by 1997 treatment failures associated with both drugs had been documented in most provinces [5,6]. Molecular epidemiology studies conducted from 2003 to 2005 in several sentinel sites throughout the Indonesia archipelago indicated widespread distribution of mutant alleles associated with resistance to CQ resistance (*pfmdr1 *86Y, *Plasmodium falciparum chloroquine resistance (pfcrt) *76T) and SP (*dihydrofolate reductase (dhfr) *and *dihydropteroate synthase (dhps)) *[7-10]. The mutant allele associated with chloroquine resistance, *pfcrt *76T, seems to be nearly fixed among the *P. falciparum *isolates collected, and *pfmdr1 *1042D alleles were mainly found in the island of Flores [9].

Previous studies of the distribution of drug resistance markers in Indonesia were not based on large-scale systematic sampling. In order to study the distribution of drug resistance markers more systematically, samples from a previously reported district wide study of seasonal malaria prevalence in West Sumba was analyzed [11]. The frequency distributions of the alleles across the villages throughout the district of West Sumba is reported.

## Methods

### Study site and sampling strategy

Sumba is a member of the Lesser Sunda Archipelago, located in the province of East Nusa Tenggara, Indonesia, at longitude 118.9 - 119.9 East and latitude 9.3 - 9.8 South, with total population of approximately 387,000 in 2007. The study design and sampling strategy have been described elsewhere [11]. Briefly, malariometric surveys were conducted twice, in March 2007 (wet season) and in August 2007 (dry season) using two-stage cluster sampling. The wet season usually occur during November to April whereas the dry season proceeds from May to October. Forty-five clusters (sub-villages) were chosen by probability proportional to size sampling. Within each sub-villages, households were chosen randomly by spinning a pointer, and were included until 100 subjects per cluster had been enrolled. Although the same 45 clusters were used in both surveys, no attempt was made to re-sample the same households; instead a new random selection of households was made within each cluster. The malaria prevalence in West Sumba was seasonal with the parasite rates was 6.83% and 4.95% in the wet and dry season, respectively. The malaria infections were mostly asymptomatic, mainly occurred among children and teenagers, and the geometric mean parasitemia decreased with age [11].

### Data collection

In each subject, blood was collected by finger or heel prick for thick and thin films on glass slides and for blood blots on filter paper (Whatman, Schleicher & Schuell, Whatman International Ltd, Maidstone, UK) for malaria diagnosis and parasite genotyping. The study was approved by Eijkman Institute for Molecular Biology Research Ethics Commission, scientific and ethical review boards of the Naval Medical Research Unit #2, and by the Indonesian National Institute of Health Research and Development.

### Genomic DNA preparation

DNA was extracted from *P. falciparum *positive blood samples diagnosed by microscopy as well as 10% of the malaria negative subjects, using chelex-100 ion exchanger (SIGMA, St Louis, USA) according to a procedure described previously [12]. DNA was either used immediately for a polymerase chain reaction (PCR) or stored at -20°C for later analysis.

### PCR amplification and genotyping of *pfcrt, pfmdr1, dhfr*, and *dhps *codons

Detection of the single nucleotide polymorphisms of *pfcrt*, *pfmdr1*, *dhfr*, and *dhps *genes was performed using PCR and restriction fragment length polymorphyms (RFLP) as described elsewhere [3,8,9,13,14].

### *Pfmdr1 *copy number

*Pfmdr1 *copy number was assessed using a Real Time PCR method [4,15,16]. The primers and a FAM-TAMRA (6-carboxyfluorescein 6-carboxy-tetra-methylrhodamine) probe specific to a conserved region of *pfmdr1 *and the primers and a VIC-TAMRA (chemical structure not released by Applied Biosystems) probe specific to β-*tubulin *were multiplexed so that both genes could be assayed in the same well. PCR reactions were performed on IQ5 Biorad^® ^and consisted of *pfmdr1 *probe (150 nmol/L), *pfmdr1 *primers (300 nmol/L), β-*tubulin *probe (100 nmol/L), β-*tubulin *primers (100 nmol/L), IQ Multiplex Powermix (1×), DNA (2-4 μL), and water up to 25 μL. The cycling conditions were: 95°C for 15 minutes for pre denaturation, and 50 cycles of 95°C for 15 seconds and 60°C for 1 minute. The cycle threshold (CT) was calculated with Optical System Biorad software ver. 1.0. DNA from strains 3D7 and W2mef were included on each plate. *Pfmdr1 *copy number was calculated according to the following formula: copy number = (E_*βtubulin*_)^CT*(βtubulin)*^/(E_*pfmdr1*_)^CT(*pfmdr1*)^. The efficiency (E) of β-*tubulin*, which was higher than that of *pfmdr1*, was assumed to be 2. *Pfmdr1*'s efficiency, relative to that of β-*tubulin*, was calculated for each plate by assuming the 3D7 control has one *pfmdr1 *copy. The additional control, W2mef, was previously determined to have three *pfmdr1 *copies [17].

## Results

### Distribution of *pfcrt *and *pfmdr1 *gene polymorphisms

PCR yielded 213 and 231 amplicons in 29 and 32 villages in the wet and dry season, respectively. The *pfcrt *76T allele was found in 92.9% of the *P. falciparum *isolates during the wet season and 84.9% during the dry season (Table 1). Analysis of isolates for the *pfmdr1 *gene revealed the existence of mutually exclusive 86Y and 1042D mutant alleles. The proportion of isolates carrying the 86Y allele was 41.9% in the wet season and 42.8% in the dry season and for the 1042D allele in the proportion were 72.8% and 53.3%. The *pfmdr1 *mutant alleles 1034C and 1246Y were not found in any of the isolates examined. Allelic combinations of 76T (*pfcrt*) with 86Y (*pfmdr1*) were found in 23.6% of the isolates during the wet season and 24.7% in the dry season whereas combinations of 76T (*pfcrt*) with 1042D (*pfmdr1*) were found in 6.2% and 5.5% in the wet and dry seasons, respectively (Table 2).

**Table 1:** Prevalence of *pfcrt*, *pfmdr1 *genes and *pfmdr1 *copy number among the isolates of *P. falciparum *in West Sumba District in dry and wet seasons

Gene	Allele	Prevalence (%)	
		Wet Season	Dry Season
*Pfcrt*	76T	92.9 (198/213*)	84.9 (147/174)
			
*Pfmdr1 *copy number > 1.5	-	10.1 (18/178)	25.9 (14/54)
			
*Pfmdr1*	86Y	41.9 (55/131)	42.8 (27/63)
	1034C	0 (0/191)	0 (0/191)
	1042D	72.8 (139/191)	53.3 (96/180)
	1246Y	0 (0/191)	0 (0/191)

* Number in bracket indicates samples examined

**Table 2:** Prevalence of allelic combinations associated with CQ and SP resistance among isolates of *P. falciparum *in West Sumba District in dry and wet seasons

	Allelic Combination	Prevalence (%)	
		Wet Season	Dry Season
CQ Resistance	76T + 86 Y	23.6 (41/174*)	24.7 (24/174)
	76T + 1042D	6.2 (11/178)	5.5 (3/54)
			
SP Resistance	108N/T + 51I + 59R	0 (99)	0 (73)
	437G + 540E	0 (92)	0 (92)

* Number in bracket indicates samples examined

### Distribution of *pfmdr1 *copy number

**Figure 1:**
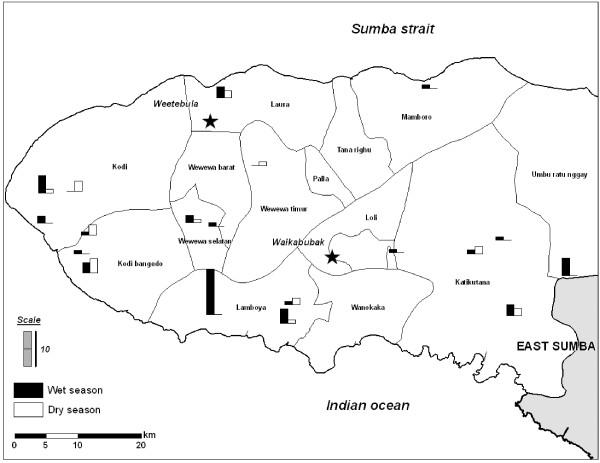
**Distribution of *pfmdr1 *gene copy number in the wet and dry seasons in West Sumba District**.

Analysis of the *pfmdr1 *gene copy number revealed 10.1% and 25.9% of the *P. falciparum *isolates in the wet and dry seasons, respectively, harbored more than one copy (Table 1). Of which, 72.2% in the wet and 85.7% dry seasons had more than one *pfmdr1 *gene copy number combined with the wild type 86N allele. The proportion of isolates that carried more than one copy number of *pfmdr1 *gene combined with 1042D was 27.8% in the wet and 14.3% in dry season, respectively. The distribution of *P. falciparum *isolates that carried more than one copy number is shown in Figure 1 in each village. The village that has *P. falciparum *isolates with the highest *pfmdr1 *gene copy number is Lamboya Dete.

### Distribution of *dhfr *and *dhps *mutant alleles

**Figure 2:**
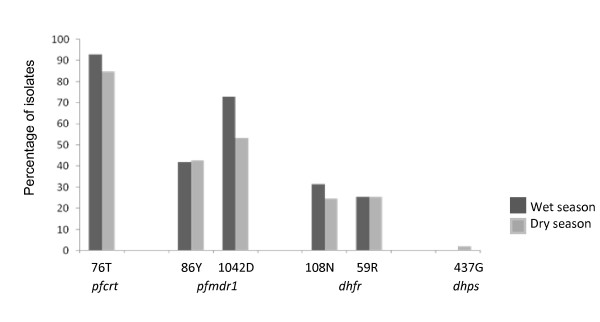
**Distribution of anti-malarial drug-resistant alleles in the wet and dry seasons in West Sumba District**. The 437G is the only mutant allele of the *dhps *gene found in this study and it was found in dry season.

Amplification of the *dhfr *gene showed that 31.5% of the isolates carried the 108N allele in the wet season whereas in dry season the proportion dropped to 24.7% (Figure 2). The 108T allele was not detected in any of the isolates examined. The 59R allele was found in 25.6% and 25.4% of the *P. falciparum *isolates in wet and dry seasons respectively, and in most cases was paired with the 108N allele. The other *dhfr *mutant alleles 16V, 50I, 51I and 164L were not found in any of the isolates examined. Amplification of the *dhps *gene revealed no mutant alleles in any of the isolates examined during the wet season whereas in the dry season, the 437G was found in 2.2% of the isolates. The other *dhps *mutant alleles (436A, 540E, 581G and 613S/T) were not detected in any of the isolates examined.

### Distribution of parasite haplotypes by village

The pattern of the parasite haplotype found in the wet and dry season is shown in Table 3. The *P. falciparum *isolates that carried wildtype alleles of the *pfcrt, pfmdr1, dhfr *and *dhps *genes, simultaneously were only found in two subjects during dry season in two remote villages of Hoha Wungo (Kodi sub-district) and Lamboya Dete (Lamboya sub-district). On the contrary, due to the very low distribution of mutant alleles of *dhps *gene, we only found two isolates that carried simoultaneous mutant alleles in the four gene during the dry season.

**Table 3:** Genotypic patterns of *P. falciparum *isolates in *pfmdr1*, *pfcrt, dhfr*, and *dhps *genes in the wet and dry seasons

Type	Genotypic Pattern*	Frequency (isolates)	
		
		Wet Season	Dry Season
1.	NNKAKASC (wild type)	-	2
2.	NN(K/T)AKASC	1	-
3.	N(N/D)TAKASC	11	25
4.	(N/Y)(N/D)TAKASC	3	12
5.	NNTAKANR	2	2
6.	N(N/D)TAKASR	2	-
7.	Y(N/D)(K/T)AKA(S/N)R	1	-
8.	YNTAKASC	19	6
9.	YNTAKA(S/N)R	2	-
10.	Y(N/D)TAKASC	15	1
11.	NDTAKASC	17	19
12.	(N/Y)NTAKANR	2	-
13.	(N/Y)(N/D)KAKASC	5	-
14.	(N/Y)(N/D)TAKA(S/N)R	3	-
15.	N(N/D)(K/T)AKASC	1	-
16.	NNTAKA(S/N)R	1	-
17.	YNTAKANR	3	-
18.	NDTAKANR	2	5
19.	NNKAKANR	4	8
20.	NNTAKASC	10	22
21.	YNTGKASC	-	2
22.	Y(N/D)TAKASC	4	1
23.	-D(K/T)AKA(S/N)C	1	-
24.	-DTAKASC	14	21
25.	-NTAKANR	2	3
26.	-NKAKASC	3	-
27.	-DKAKASC	2	4
28.	-NTAKASC	2	5
29.	-(N/D)TAKA(S/N)C	1	-
30.	-(N/D)TAKASC	10	24
31.	-(N/D)KAKASC	1	1
32.	-DTAKA(S/N)C	2	2
3.	(N/Y)(N/D)TAK---	5	2
34.	(N/Y)(N/D)KAK---	5	2
35.	(N/Y)(N/D)TAK---	8	2
36.	NDTAK---	7	2
37.	NNTAK---	8	3
38.	YNT-----	6	2
39.	YNK-----	1	1
40.	-(N/D)TAK---	1	2
41.	-DTAK---	5	3
42.	-(N/D)(K/T)-----	1	3
43.	N-AK----	2	2
44.	ND------	10	22
45.	--T-----	8	20
			
	Total	213	231

*1^st ^letter = N86Y and 2^nd ^letter = N1042D for *pfmdr1 *gene, 3^rd ^letter = K76T for *pfcrt *gene, 4^th ^letter = A437G and 5^th ^letter = K540E for *dhps *gene, 6^th ^letter = A16V, 7^th ^letter = S108N/T and 8^th ^letter = C59R for *dhfr *gene. Letters in parentheses indicate mixed allelic infection. -- indicates unsuccessful amplification.

## Discussion

Molecular analysis of the *P. falciparum *isolates collected throughout the district of West Sumba indicated that the 76T allele of the *pfcrt *gene, a molecular marker for CQ resistance, has almost gone to fixation in the local population. Furthermore, nearly half of isolates also simultaneously carried *pfmdr1 *mutant alleles, either as 86Y or 1042D (Figure 2). Five mutant alleles of the *pfmdr1 *gene have been implicated in CQ resistance: 86Y, 184F, 1032C, 1042D and 1246Y. Of these, 86Y and 1042D are commonly found throughout Asia and Oceania whereas the 1032C and 1246N alleles are typical in *P. falciparum *isolates of Latin American origin [18,19]. In Indonesia, the 86Y allele is more prevalent in western provinces whereas the 1042D allele dominates in the east. Interestingly, these alleles were found to be mutually exclusive [9]. These findings endorse abandonment of CQ as the first-line treatment of uncomplicated *P. falciparum *in the district of West Sumba.

Previous data in Africa suggest an associaton between amodiaquine (AQ) treatment failure and parasite isolates carrying a combination of *pfcrt *76T and *pfmdr1 *86Y alleles [19,20]. In this study *P. falciparum *isolates frequently carried the allelic combination of *pfcrt *76T and *pfmdr1 *86Y, but previous study showed artesunate (AS) and AQ combination therapy to be highly effective [10]. This finding suggests that the parasite haplotype 76T and *pfmdr*1 86N is still sensitive to AQ treatment, particularly in combination with AS. Otherwise, the high efficacy of AQ might be associated with the absence of 1246Y allele of the *pfmdr1 *in any of the isolates examined. Previous studies found out the selection of *pfmdr1 *1246Y alleles following AQ treatment [21-23].

In the present study, the distribution of the *Pfcrt *76T, *pfmdr1 *86Y, *dhfr *108N and *dhfr *59R alleles were slightly higher in the wet season (Figure 2). It may be suggested that this overrepresentation of the drug-resistant alleles may reflect relatively higher drug pressure during in the wet season. This suggestion is supported by the fact that the wild type *P. falciparum *isolates that is found during the dry season.

Analysis of the *pfmdr1 *gene copy number revealed over 10% and 25% of the *P. falciparum *isolates harbor more than one copy in wet and dry season, respectively. This finding is similar to results in Thailand where the increase in the *pfmdr1 *gene copy number was associated with resistance to mefloquine and lumefantrine [4]. This finding may indicate that mefloquine and lumefantrine, which are currently used as partner drugs for artesunate and arthemeter, may not be suitable for use in Sumba. Previous studies also reported that increased in copy number of the *pfmdr1 *gene is more often usually paired with wildtype 86N allele in comparison to the mutant 86Y [4,19]. This phenomenon is also observed in this study where the 1042D was the most common *pfmdr1 *allele found. The increase in *pfmdr1 *copy number was more often found along with either 86N or 1042N in comparison to 86Y of 1042D. The findings indicate that under CQ pressure the parasite may undertake either the *pfmdr1 *gene amplification or mutations, and that the former usually takes place earlier as indicated in the previous study [24].

The proportion of isolates carrying mutant alleles of the *dhfr *and *dhps *gene are relatively small in comparison to the *P. falciparum *isolates collected from the other parts of Indonesia [7-9]. For the *dhfr *gene, the majority of isolates carried the double mutation 108N+59R, whereas for *dhps *only two isolates carried the 437G allele. It follows that the use of SP as an interim alternative option to AS-AQ could be considered in this area, as the majority of the *P. falciparum *isolates are likely still sensitive to the drug. Treatment failure with SP in Africa is associated with the presence of quintuple mutations in *dhfr *and *dhps *genes, respectively [25].

This study shows that the determining mutant allele associated with CQ resistance is nearly fixed in the parasite population found in the district of West Sumba. A high prevalence of *pfmdr1 *mutant alleles was also found, reinforcing that CQ should no longer be used in this area. Except for the mutant dhps 437G allele which was only detected in two subjects during dry season, the distribution of the other drug-resistant alleles is slightly higher in the wet season. In addition, the increasing number of *P. falciparum *isolates carrying more than one copy number of *pfmdr1 *limit candidate partner drugs for artemisinin as this mutation is associated with resistance to mefloquine and lumefantrine, two of the compounds that are currently used in ACT. However, the relatively low prevalence of mutant alleles in the *dhfr *and *dhps *genes is encouraging for future ACT formulations in this area.

## Competing interests

The authors declare that they have no competing interests.

## Authors' contributions

PBSA, WOR, AIS, AR, EIR, MAK and DS performed field samples collection, molecular assays, data analysis, and the manuscripts writing. K, ST, RMD, FNC, and AS collected field samples and performed data analysis. AJAM and RWS contributed to data analysis, and the manuscript writing. DS, WOR, AJAM and RWS design the study and were responsible for management and fund raising for this study. All authors read and approved the manuscript.
